# Whole Genome Sequencing of the Moruga Hill Rice (*Oryza glaberrima*) Reveals Its African Ancestry and the Presence of Candidate Stress-Tolerance Genes

**DOI:** 10.3390/biotech15040073

**Published:** 2026-08-25

**Authors:** Uddesh M. Sahadeo, Omar Ali, Adesh Ramsubhag, Christine Carrington, Arianne Brown Jordan, Jayaraj Jayaraman

**Affiliations:** 1 Plant-Microbe-Biotechnology Laboratory, Department of Life Sciences, Faculty of Science and Technology, The University of the West Indies, St. Augustine 311126, Trinidad and Tobago; uddeshsahadeo987@live.com (U.M.S.); omarali0418@gmail.com (O.A.); adesh.ramsubhag@uwi.edu (A.R.); 2 Department of Preclinical Sciences, Faculty of Medical Sciences, The University of the West Indies, St. Augustine, Trinidad and Tobago; christine.carrington@uwi.edu (C.C.); arianne.brown-jordan@uwi.edu (A.B.J.)

**Keywords:** *Oryza glaberrima*, rice, Moruga Hill Rice, whole genome sequencing, genomics, phylogenomics

## Abstract

Moruga Hill Rice (MHR) is an African rice (*Oryza glaberrima* Steud.) brought to Trinidad by formerly enslaved African Americans and has been grown for many generations in Trinidad at subsistence and commercial scale. Despite its historical and agricultural significance, genomic resources specific to MHR remain unexplored, and its genetic composition, evolutionary history, and potential agronomic traits have not been characterized. This current study presents the first draft genome assembly of the MHR genome using a hybrid sequencing approach. The MHR genome size was found to be ~372.9 Mb with 56,073 predicted genes. Variant analysis revealed a total of 3,318,242 variants, of which 2,440,476 were SNPs, and 877,766 were InDels. Several candidate genes encoding proteins with orthology to previously characterized biotic resistance and abiotic stress-responsive genes in rice were identified. Potential gene families identified prompt further investigation of their roles in MHR drought and salt stress responses. Phylogenomic analysis of *O. glaberrima* landraces suggests that MHR shares close genetic affinity with the IRGC−104595 Malian landrace, consistent with historical records. This assembly thus expands the African rice genomic repository, providing a foundation to understand the genetic architecture underlying key phenotypic traits and identifying potential novel gene sources in MHR for rice improvement in the Caribbean region.

## 1. Introduction

*Oryza glaberrima* was transported to the Caribbean during the Trans-Atlantic Slave Trade in the mid-17th century but domesticated approximately 3500 years ago [1]. Moruga Hill Rice (MHR) was later introduced to Moruga, Trinidad, by the “Merikins” (African American ex-slaves) [2] who served in the British Corps of Colonial Marines during the War of 1812—and subsequently adopted and maintained by Warao-descendant First Peoples [3,4,5]. For more than two centuries, MHR has remained a culturally important dietary staple cultivated by local communities [6,7]. Hill rice types grown in the Caribbean closely resemble West African landraces in both morphology and physiology. For instance, Surinamese “black rice” has been shown to be genetically similar to an Ivory Coast *O. glaberrima* landrace [8], suggesting that Caribbean hill rice varieties, including MHR, may share similar origins.

Domesticated *O. glaberrima* is recognized for its resistance to major rice diseases. African landraces often carry resistance (*R*) genes such as members of the *Xa* group and *Pi-ta*, which confer resistance to bacterial blight caused by *Xanthomonas oryzae* pv. *oryzae* and rice blast caused by *Magnaporthe oryzae*, respectively [9,10]. These diseases can result in yield losses of 20–50% and up to 65% in susceptible varieties [11,12]. In addition, African landraces possess tolerance (*T*) genes associated with adaptation to abiotic stresses. Compared with many wetland *O. sativa* cultivars, *O. glaberrima* also demonstrates strong weed competitiveness due to vigorous early growth, efficient root systems, and rapid maturation [13]. Given its probable West African origin and more than two centuries of geographic isolation and farmer selection in Trinidad, MHR represents a genetically distinct and unexplored reservoir of novel alleles for stress tolerance and disease resistance that could broaden the narrow genetic base of cultivated *O. glaberrima* improvement.

Despite these advantages, upland *O. glaberrima* types generally exhibit lower productivity and are less suited to large-scale cultivation. Yield reductions are associated with lodging due to weak culms, panicle shattering, and susceptibility to environmental conditions such as strong winds [14]. Genomic analysis may also reveal the existence of loci associated with homology to agronomically relevant genes. Moreover, given the lengthy time frame since its arrival in Trinidad, potential uncharacterized gene models may also be attributed to ongoing gene loss/gain through evolutionary time [15]. To date, the genome of the MHR has neither been sequenced nor has any analysis of genomic characteristics been reported to investigate its origins and putative agronomic genes. Given its nutritional, cultural, and genetic significance, genomic analysis is essential for its conservation and utilization in molecular breeding initiatives.

In this study, the MHR genome was sequenced and assembled for the annotation of genes, transcription factors, and repeat elements, and to identify putative candidate disease resistance and stress tolerance genes based on homology and functional ontologies. Genetic variant analysis was conducted to further characterize the genome in comparison to the *O. glaberrima* reference assembly (GCF_000147395.1). Nucleotide-binding leucine-rich repeat (NLR) genes were characterized and compared with known blast and blight resistance genes using phylogenetic and sequence homology analyses. Phylogenomic analysis was also performed to shed light on the relationship of MHR to 112 African rice accessions and determine its closest ancestral origins.

## 2. Materials and Methods

### 2.1. Plant Material Sampling

For this study, seeds of MHR from Moruga, Trinidad and Tobago, were provided by HRM Grand Chief of the First Peoples Sovereign Nations, Eric Lewis. Seeds were propagated through successive generations from a single seed source to increase seed availability while maintaining the original germplasm identity. Seeds were soaked in sterile distilled water for 24 h and pre-germinated on moistened filter paper for 48 h in darkness [16]. Germinated seeds were sown in seedling trays containing a 1:1 peat moss–clay soil mixture for 2 weeks before transplanting into 2-gallon pots filled with clay soil collected from rice fields in Moruga, Trinidad. Plants were grown to maturity, and seeds were harvested for three consecutive propagation cycles. During propagation, multiple individuals were maintained, and seeds were bulk harvested from representative plants to minimize genetic drift and maintain the genetic diversity of the accession. A voucher specimen was deposited at the National Herbarium of Trinidad and Tobago, The University of the West Indies, St. Augustine (Uddesh Sahadeo 1, (TRIN [Barcode 005732])). Seeds from the third propagation cycle (F_3_) were surface sterilized, pre-germinated on sterile water-moistened filter paper, and grown in pots containing Moruga field soil for 21 days. Terminal leaves from a single selected plant were harvested, placed on dry ice, and transported to the laboratory for DNA extraction.

### 2.2. DNA Extraction

Young leaf tissue was flash frozen in liquid nitrogen within 30 min of sampling and ground to a fine powder using a sterilized mortar and pestle. Genomic DNA was extracted using a CTAB protocol (2% CTAB, 1% PVP, 100 mM Tris-HCl, 1.4 M NaCl) [17] followed by phenol–chloroform purification [18]. DNA was eluted in 50 µL TE buffer (10 mM Tris-Cl, 1 mM EDTA, pH 8.5), treated with 5 µL RNase A (10 mg/mL), and incubated at 37 °C for 20 min. Quantification and purity were assessed using a Jenway Genova Nano spectrophotometer (Jenway, Staffordshire, UK), and integrity was verified via electrophoresis on a 1% TAE agarose gel.

### 2.3. Illumina Library Preparation and Sequencing

A Bioinformatic pipeline highlighting key steps taken for whole genome assembly and genomic analysis is presented in Figure S11. Illumina sequencing was performed by Novogene (Sacramento, CA, USA). A 150 bp short-insert genomic library was prepared using the NEBNext Ultra DNA Library Prep Kit for Illumina (New England Biolabs, Beverly, MA, USA) and sequenced on the NovaSeq X Plus platform (150 bp paired-end). Raw reads were filtered to remove adapter contamination, reads with >10% Ns, and reads with ≥50% low-quality bases (Phred < 5).

### 2.4. Oxford Nanopore Technologies (ONT) Library Preparation and Sequencing

ONT libraries were prepared using the ligation sequencing kit (SQK-LSK109; ONT, Oxford, UK) with minor modifications. Three µg of genomic DNA was used for library preparation. DNA end-prep was performed using the NEBNext Ultra II End Repair/dA-Tailing Module. Adapter ligation and clean-up were performed using Long Fragment Buffer to enrich fragments > 3 kb. Sequencing was conducted on an ONT GridION^®^ X5 (Oxford, UK) at the Department of Preclinical Sciences, Faculty of Medical Sciences, the University of the West Indies, Trinidad and Tobago. Twelve µL of library was loaded across two FLO-MIN106 (v9.4.1) flow cells. Raw signals were recorded using MinKNOW (v23.11.7) [19], and base calling was performed with Guppy (v6.3.8) [20] using a minimum Q-score = 9. A 3.6 kb DNA CS control included during library preparation was removed by aligning reads to the control sequence using minimap2 (v2.28) [21], followed by extraction of unmapped reads using *samtools* (v1.20) [22].

### 2.5. Genome Assembly, Polishing and Scaffolding

Paired-end Illumina read quality was assessed using FastQC (v0.12.1) [23] and filtered with fastp (v0.23.4) [24] (Q ≥ 30), including polyG trimming and overlap correction. ONT reads from both flow cells were merged and processed using NanoFilt [25] (v2.8.0; mean Q ≥ 9) and Porechop (v0.2.4) [26] for adapter removal. Error correction was performed with Canu (v2.2) [27] (genome size estimate = 381 Mb). De novo assembly was performed using Flye (v2.9.3) [28] with five polishing iterations. Haplotigs and overlaps were purged with purge_dups (v1.2.5) [29] using the output of minimap2 (v2.28) [21]. Cutoff thresholds were based on the read coverage histogram output with a singular mean histogram peak = 23. Therefore, the reduced thresholds, -l = 5, -m = 18, -u = 54, were used for haplotig removal to generate a collapsed haploid assembly. Error-corrected ONT reads were mapped to the assembly using minimap2, followed by polishing with one round of Racon (v1.5.0) [30] and one round of Medaka (v1.12.1) [31]. Illumina reads were mapped to the Medaka-polished assembly using minimap2 [21] and samtools [22]. Three iterative rounds of polishing were performed using HyPo (v1.0.3) [32], remapping reads after each iteration. Final polishing and gap filling were conducted using the ntEdit+Sealer pipeline (*ntHits* v0.0.1, *ntEdit* v1.3.5, *ABySS* v2.3.2) [33] with *k*-mers of 65, 55, 47 and 40, and a 40G ABySS-bloom filter. Reference-guided scaffolding against the *O. glaberrima* reference genome (GCF_000147395.1) was performed using RagTag (v2.1.0) [34]. Pseudomolecules were extracted individually, and unplaced contigs were concatenated as ChrUn. *BUSCO* analysis (v5.5.0) [35] was performed using the embryophyta_odb10, liliopsida_odb10 and poales_odb10 datasets. Long transposable repeat (LTR) Assembly Index (LAI) score was calculated using *LTR_retriever* [36,37] to assess the genome assembly contiguity. The assembly consensus quality value (QV) was determined using *Merqury* [38]. *K*-mer analysis was performed using Jellyfish [39] (v2.3.1) and Genomescope (2.0) [40] to calculate the heterozygosity rate at *k*-mer = 19. Assembly statistics were generated using bbmap (v39.06) [41] and *QUAST* (v5.2.0) [42].

### 2.6. De Novo Assembly of Unmapped MHR Genome and Annotation of Genes

High-quality Illumina paired-end reads were mapped to the GCF_000147395.1 genome using *bwa-mem2* (v2.2.1) [43]. The BAM alignment file was split into mapped and unmapped reads using BAMTools (v 2.5.2) [44], and unmapped reads were de novo assembled with ABySS (v2.3.2) [45] (k-mer = 65).

### 2.7. Genome Annotation

Repeats within pseudomolecules and unplaced contigs were masked using RepeatMasker [46] within OmicsBox (v3.1.11) [47] with the RMBlast search engine using the Dfam consensus repeat database (release 3.7) and species set to *O. glaberrima*. Repeat annotation of the unmapped assembly was also performed but analyzed separately from the main genome assembly. *Augustus* (v3.5.0) [48] was used for gene prediction with *Oryza* as the model using extrinsic evidence (*O. glaberrima* reference cDNA and protein sequences). Functional annotation was performed using *BLASTx* (v2.16.0) against the NCBI NR database (March 2024), InterProScan (v5.52) [49], and GO terms were assigned using *BLAST2GO* (v6.0.3) [50] with the GOA database (version date 2024.05). Transcription factors were predicted using PlantTFDB 4.0 [51].

### 2.8. Transposable Element Annotation

A de novo TE library was generated using the EDTA [52] pipeline (species = ‘rice’). CDS sequences from Ensembl Genomes release 59 were used to mask coding regions. A manually curated rice TE database (rice7.0.0.liban) was used (https://github.com/oushujun/EDTA/blob/master/database, accessed on 29 March 2024) [52]. The various TE classes, counts, and respective masked sizes were all recorded.

### 2.9. Phylogenetic Analysis of MHR NLR Genes with Cloned R Genes for Disease Resistance

Canonical NLR genes were identified from predicted proteins using the *Resistify* [53,54] pipeline with default parameters. Resistify incorporates gene model structure and domain validation and yields a canonical set of full-length NLR candidates. Consequently, the latter was considered more appropriate for phylogenetic inference, which requires aligned, evolutionary comparable sequences [55]. Phylogenetic analysis was performed using Resistify-identified NLRs due to their significantly high recall scores for NLR genes across multiple plant species, particularly in rice [56]. ClustalW alignment [57] was performed using MEGA (v11) [58] on the amino acid sequences of the NB-ARC domains and a series of cloned NLR R genes known to confer blast and blight disease resistance in rice (Tables S1 and S2, Supplementary File S1). Maximum likelihood trees were constructed using RAxMLGUI (RAxML v8.2.12) [59] with an MRE bootstrap parameter. Filtered reciprocal best BLAST hit (RBH) tests were carried out on MHR protein sequences for confirmation of NLR orthology. Minimum identity for matches was set to 70%, minimum query coverage was set to 50%, and the E-value cutoff = 1.0 × 10^−5^.

### 2.10. Genomic Variant Analysis of the MHR Genome Assembly

Illumina reads were mapped to the GCF_000147395.1 reference using *bwa-mem2* (v2.2.1) [43]. Variants were called using GATK HaplotypeCaller (v4.2.3.0) [60] with duplicate reads being removed, and base quality recalibration was performed using GATK BaseRecalibrator and ApplyBQSR. Filtering was performed using the following criteria: MQ < 50.0, DP < 10.0 and QD < 10.0. The applied filtering thresholds were selected to retain high-confidence variants while minimizing false-positive calls. Low MQ variants were excluded because they are often associated with ambiguous read alignments in repetitive or duplicated genomic regions. Variants with low DP were removed due to insufficient sequencing support, which increases the likelihood of stochastic sequencing errors or unreliable genotype assignment. Similarly, low QD variants were excluded because they indicate poor variant confidence relative to the read depth supporting the call. Together, these filters improve the reliability of downstream population genetic and functional analyses. Variants were annotated using SnpEff (v5.2) [61] with *O. glaberrima* annotations from Ensembl BGMPP (release 32).

### 2.11. Phylogenomic Analysis to Estimate the Origins of the MHR

Whole genome paired-end sequences from 111 *O. glaberrima* accessions [62,63] and the Surinamese “black rice” accession TVA6749, as used in a previous study [8] (Table S4), were obtained from the SRA database. Reads were mapped to the reference genome using bwa-mem2 [43] and processed using samtools [22]. SNPs were called using the GATK Best Practices workflow and filtered for chromosomes 1–12. Variants with MQ < 50.0, DP < 10.0, and QD < 10.0 were excluded to remove low-confidence SNPs arising from poor read alignment, insufficient sequencing support, or weak variant quality relative to sequencing depth, while MAF ≥ 0.05 and missing rate ≤ 10% were applied to retain high-confidence, phylogenetically informative variants and minimize bias from incomplete genotype data. Linkage disequilibrium (LD) pruning was carried out using a sliding-window approach implemented in PLINK [64] (--indep-pairwise 50 10 0.2) to retain a set of independent SNP markers. A total of 62,646 SNPs across 113 samples were retained and converted to FASTA using VCF-Kit (v0.3.0) [65]. Nucleotide diversity (π) and Nei’s estimation of heterozygosity were calculated using VCFtools [66]. Maximum likelihood tree analysis was performed using RAxMLGUI [59] using the rapid bootstrap method with replicates of 1000 with the K2P + GAMMA model (best-fitting substitution model). Principal component analysis (PCA) was performed in R using eigenvectors generated in PLINK [64], and the top two significant PCs were used to create a two-dimensional PCA plot. Ancestry coefficients were determined using sNMF [67] (K = 1–10) in R with the LEA package [68].

## 3. Results

### 3.1. Genome Sequencing of the Moruga Hill Rice

Long-read sequencing of the MHR was performed using the ONT platform, and two (2) flow cells produced 1,366,700 reads constituting 11.49 Gbp (~33× genome coverage) (Table 1). ONT read length N50 was recorded at 15.55 kbp and 13.02 kbp for the respective flow cells with a minimum cutoff Q-score = 9. Paired-end short-read sequencing performed using the Illumina sequencing platform produced 317,401,980 reads comprising 47.60 Gbp (~137× genome coverage). The MHR genome assembly recorded an LAI of 9.85 while *k*-mer analysis estimated a QV score of 32.73, thus reflecting a fairly strong nucleotide accuracy for its draft-quality assembly [36].

### 3.2. De Novo Assembly of the MHR Genome

De novo assembly was performed using ONT reads, followed by genome polishing with high-quality Illumina reads. The impact of polishing the long-read assembled genome was evaluated by comparing the mapping rate, properly paired rate, mismatch/indel profiles and depth distribution (Table 2; Figures S5 and S6). The final polished assembly was 372.9 Mb, consisting of 1333 contigs with a contig N50 of 153 kbp (Table 3). Reference-guided scaffolding generated 12 pseudomolecules totalling 379.7 Mb, with 130 contigs (2.2 Mb) remaining unplaced (Table 3). Genome contiguity based on the scaffold N50 length was found to be 6 Mbp. The total genome gap represented 1.794% of the assembly, with chromosome 12 containing the largest gap (~1.08 Mb) (Table 4). *BUSCO* analysis of the final assembly indicated high genic completeness, with 98.9%, 96.7%, and 97.4% complete genes based on the Embryophyta, Liliopsida, and Poales datasets, respectively (Figure S1, Supplementary File S1). The MHR genome heterozygosity rate was estimated to be 0.0915% using the *k*-mer method (Figure S2, Supplementary File S1).

### 3.3. Annotation of the MHR Genome Assembly

Extrinsic evidence-guided ab initio gene prediction of the repeat-masked MHR genome assembly and unmapped-reads de novo assembly identified a total of 61,486 predicted genes. A total of 56,073 genes were identified, of which 54,102 were functionally annotated using the NCBI non-redundant (NR) database (6 October 2024). Protein domain annotations were assigned using *InterProScan* with the SUPERFAMILY, PANTHER, and Pfam databases (Table S5, Supplementary File S1). Gene Ontology (GO) terms were assigned to ~75% of the total predicted genes.

### 3.4. Transcription Factor Prediction

PlantTFDB prediction tool identified 1790 transcription factor (TF) genes belonging to 56 TF families (Figures S3 and S4; Tables S6 and S7). The seven most abundant TF families accounted for 47.04% of all TFs: bHLH (153), NAC (148), ERF (136), MYB (119), C2H2 (105), WRKY (99), and bZIP (82).

### 3.5. Transposable Element Composition of the MHR Genome Assembly

The repeat element content was 50.35% of its masked genome (~187.7 Mb), with chromosome 1 possessing the largest proportion of transposable elements (TEs) at 5.04% (Table S8). The largest class of TEs belonged to long terminal repeat retrotransposons (LTR-RTs), followed by terminal inverted repeat (TIR) sequences, forming 23.63% and 18.54% of the MHR genome, respectively (Table 5). Approximately 17.97% of the TEs were classified as miniature inverted-repeat TEs (MITEs). Of all retrotransposons, LTR/*gypsy* represented the highest proportion at 19.36% of the masked genome, while LTR/*copia*, LTR/*TRIM*, and LTR/*unknown* were 3.62%, 0.16%, and 0.49%, respectively. Among the non-LTR retrotransposons, LINE/*unknown* was the greatest at 1.36% of the masked genome.

### 3.6. Phylogenetic and Reciprocal BLAST Analysis of MHR NLR Genes

**Figure 1:**
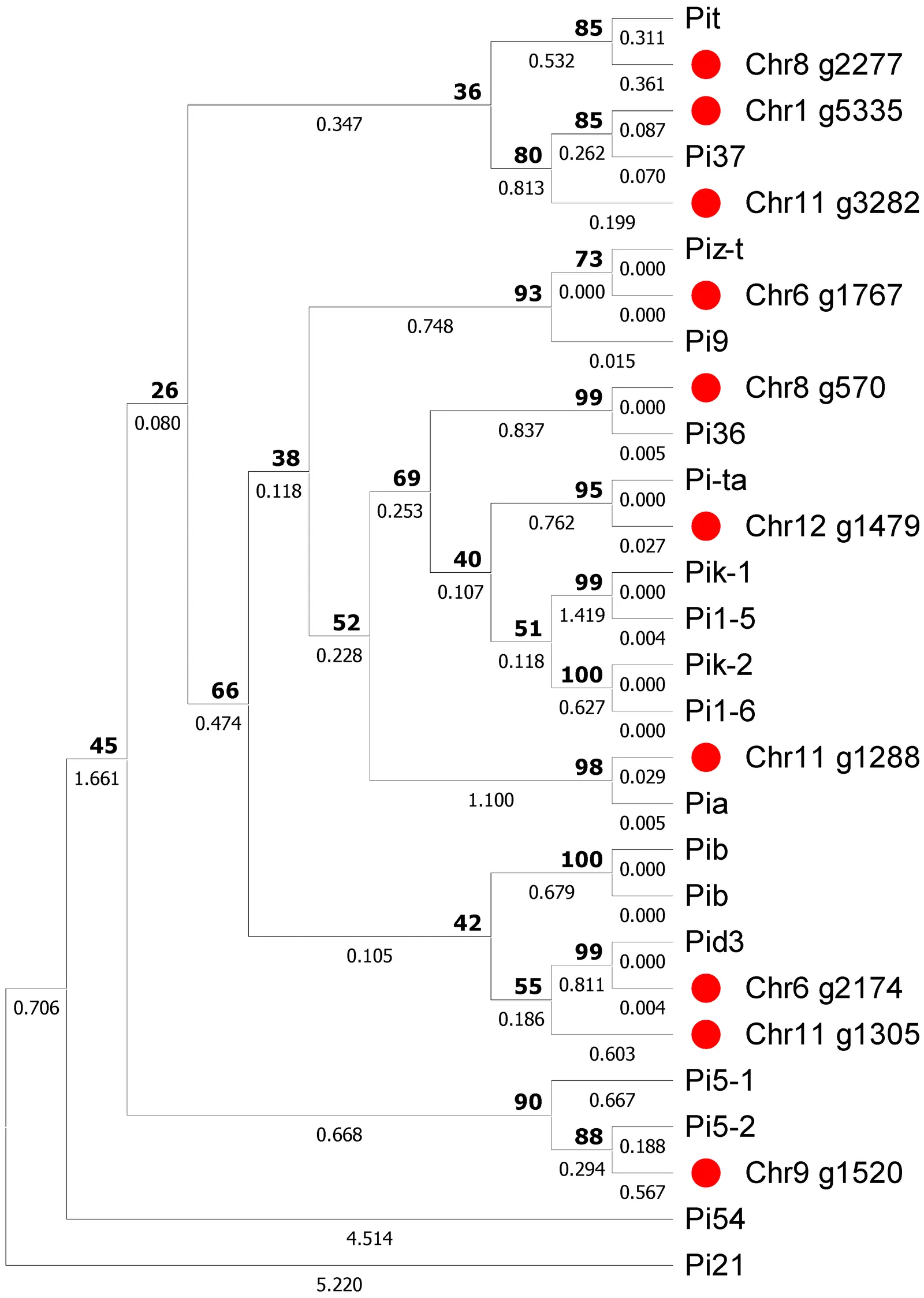
Phylogenetic relationships of MHR NLR genes and cloned blast disease resistance genes. Maximum-likelihood phylogenetic tree showing relationships among NB-ARC domains from known rice resistance genes and NLR genes identified in the MHR genome. A total of 1000 bootstrap iterations were performed. MHR genes are indicated by red circles, bootstrap support values are shown above nodes, and branch lengths shown below nodes represent genetic distances.

*Resistify* identified 474 canonical NLR genes, with chromosome 11 containing the highest NLR counts (105) (Figure S7; Tables S9 and S10). Most genes belonged to the CNL (218) and CN (173) classes. No TNL genes were detected. Additional classifications included TN (5), NL (48), and N (30). To assess the relationships between MHR NLR genes and known blast resistance genes, we constructed a maximum-likelihood phylogeny of NB-ARC domains from ten MHR-derived NLR proteins and fourteen previously cloned rice blast resistance proteins, with node support assessed using 1000 bootstrap replicates (Figure 1, Figures S8 and S9). Four MHR proteins clustered with strong bootstrap support (BS ≥ 95%) alongside known resistance proteins: Chr12_g1479 and Chr8_g570 formed a well-supported clade (BS = 99%) sister to Pi-ta (BS = 95%), within a broader subclade that also included Pi36. Chr11_g1288 was grouped with Pia (BS = 98%) and Chr6_g2174 clustered with Pid3 (BS = 99%). In contrast, several proteins showed only moderate or weak support for their placement. Chr6_g1767 grouped with the Pi9/Piz-t clade at reduced support (BS = 73%); Chr9_g1520, Chr11_g3282, and Chr1_g5335 fell within the broader Pi5/Pi37 lineage (BS = 80–88%); and Chr11_g1305 (BS = 55%) and Chr8_g2277, which occupied an isolated long branch near the deepest tree split, showed the weakest support for any specific relationship with the cloned resistance proteins.

To independently corroborate these placements, we performed reciprocal best BLAST hit (RBH) analysis of MHR NLR proteins against previously characterized rice resistance protein sequences (Table 6). This analysis confirmed high-confidence orthology for two genes that also met the reviewer-specified >85% similarity threshold together with strong bootstrap support: Chr11_g1288 shared 92.02% similarity with Pia-C_2 (99% coverage, bit score 1840), consistent with its phylogenetic placement sister to Pia (BS = 98); and Chr6_g2174 shared 96.54% similarity with Pid3 (100% coverage, bit score 1819), consistent with its clustering with Pid3 (BS = 99). Chr8_g570 showed 85.37% similarity to Pi36 by RBH, placing it just at the high-confidence threshold and consistent with its phylogenetic position within the Pi-ta/Pi36 subclade. By contrast, Chr1_g5335 fell just below the similarity threshold (84.44% to Pi37, with reduced alignment coverage of 74%), corroborating its designation as a moderate rather than high-confidence homolog based on the phylogenetic analysis alone. Together, the convergence of phylogenetic and RBH evidence for Chr11_g1288 and Chr6_g2174 provides strong support for their classification as candidate orthologs of *Pia* and *Pid3*, respectively, while the remaining MHR NLR genes represent more distantly related members of the rice resistance gene repertoire.

RBH analysis additionally identified twelve further MHR proteins with homology to previously characterized resistance genes not included in the NB-ARC phylogeny, including several with high similarity and full-length coverage: Chr8_g4137 (100% similarity to xa13), Chr11_g3558 (100% to Xa23), Chr6_g2238 (99.76% to Pik-2), Chr4_g2509 (98.38% to Pi21), and Chr12_g2279 (97.30% to Xa25) (Table 6). These included homologs of both blast (*Pi*) and bacterial blight (*Xa*) resistance genes, suggesting the MHR genome harbors a broader repertoire of candidate disease resistance loci beyond those captured in the NLR-focused phylogenetic analysis.

### 3.7. Mining of Defense and Stress-Response Associated Genes in the MHR Genome

**Figure 2:**
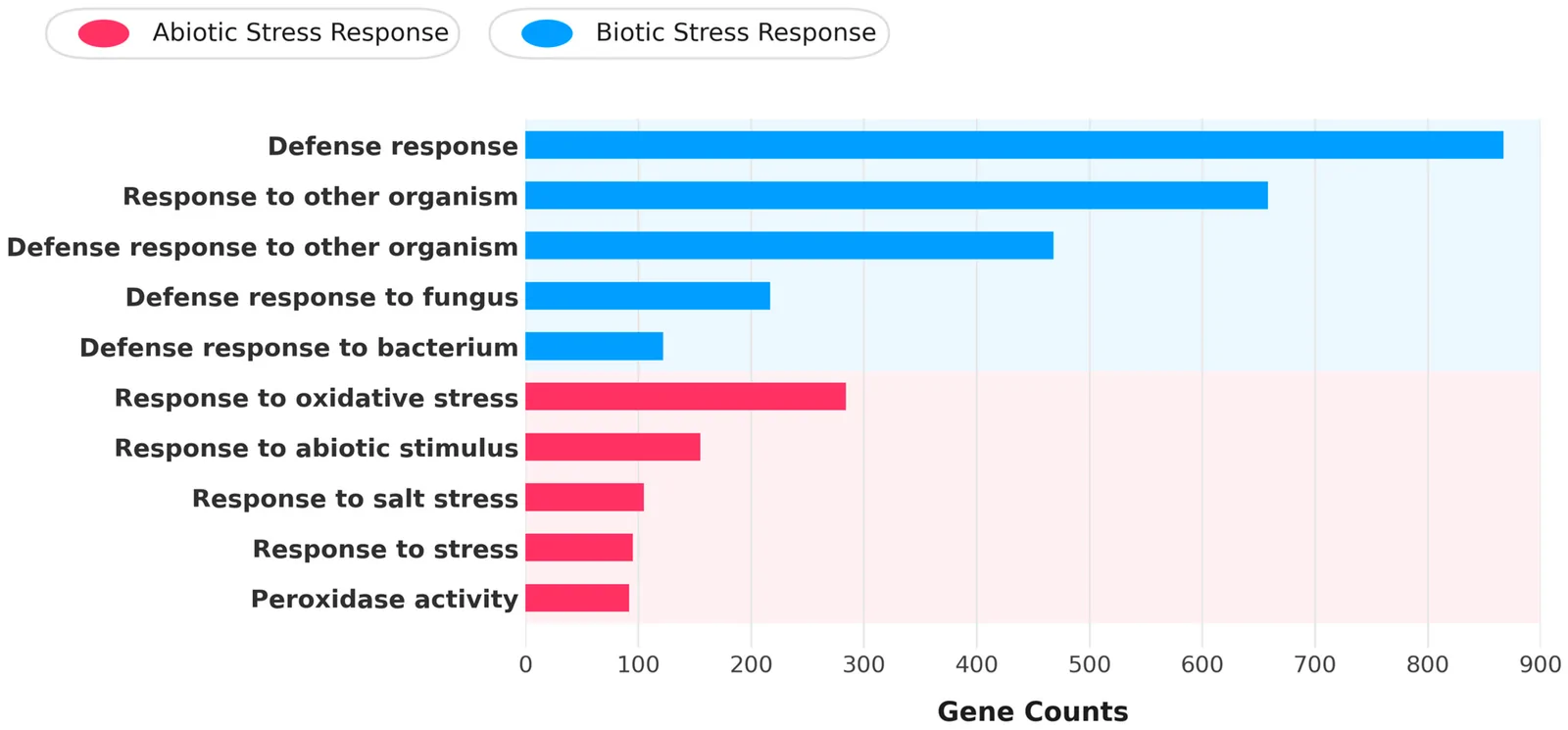
Gene ontology (GO) analysis of stress response-related genes in the MHR genome. The top five GO biological process terms associated with biotic (blue) and abiotic (red) stress responses are shown based on the number of annotated genes assigned to each category. Gene counts indicate the representation of candidate stress- and defense-associated MHR genes.

Gene annotations were screened using 40 GO terms associated with disease resistance and abiotic stress (Figure 2; Table S3). A total of 2626 genes were associated with defense responses and were assigned to biological process GO terms including “defense response”, “defense response to bacterium”, and “defense response to fungus”, with 865, 124, and 217 genes, respectively. Abiotic stress screening across 14 GO terms identified 1116 genes, with chromosomes 1, 3, 4, 6, and 10 containing the highest numbers of stress-associated genes. Major categories included “response to oxidative stress” (285 genes), “response to salt stress” (106), and “peroxidase activity” (93). Other notable ontologies included desiccation (60), water deprivation (37), heat (87), and cold (57).

Multiple stress-related TFs were identified, which include specific heat shock protein (HSP) members from groups A and C, such as A-2b, A-6a, rHsf13/C-1a, C-1b, A-1, A-2a, A-2c, A-2e, A-5, Hsf3, C-2a, and C-2b. Various HSPs, including 23.2 kDa HSP, chloroplastic HSP90-5, mitochondrial HSP90-6, HSP82, HSP81-1, HSP81-2, and 70 kDa HSP, as well as several small HSPs (sHSPs) from classes I, II, III, and VI, as well as mitochondrial HSPs, were also identified across the genome. Numerous gene families throughout the MHR encoded for homologs of abscisic acid (ABA) receptors (Pyrabactin Resistance-like (PYL) family), dehydration-responsive element-binding (DREB) transcription factors, aquaporins (plasma membrane intrinsic protein 1-1 (PIP1-1), PIP1-2, and PIP1-3), dehydrins (such as responsive to ABA (Rab) proteins), various signalling components such as calcium-dependent protein kinases (CPKs), MAP kinases (MAPKs), superoxide dismutase (SOD), and 6-phosphogluconate dehydrogenase. Cold-response genes included calmodulin-binding (CaM-binding) receptor-like cytoplasmic kinase 2, CDPK11 and CDPK13, MYB4, WRKY45, and thioredoxin H1. A substantial number of peroxidases are annotated throughout the MHR genome with peroxidase activity and oxidative stress response ontologies.

### 3.8. Genome Variant Analysis of the MHR Genome Assembly

A total of 3,318,242 variants were identified in the MHR genome, comprising 2,440,476 SNPs and 877,766 InDels (Table S11) with an average density of 7.25 SNPs/kb and 2.31 InDels/kb, respectively. The SNP density (SNPs/kb) across all chromosomes varied to a small degree. Chromosome 9 recorded the highest density at 5.76 SNPs/kb, while chromosome 5 had the lowest at 6.85 SNPs/kb. Chromosome 12 had the highest density of InDels at 2.37 InDels/kb, while chromosome 9 had the lowest at 1.99 InDels/kb. Overall, 94.94% of variants were homozygous, while 4.54% were classified as heterozygous with a Transition/Transversion (Ts/Tv) ratio of 2.3168. Mutations were also classified by their putative effects based on impact, with ~3.27% found to be low impact, ~3.66% moderate impact and ~0.41% high impact (Table S12). When grouping effects into functional classes, non-sense variants represented the lowest proportion at 0.59%, while missense and silent variants were 53.58% and 45.85%, respectively. A total of 55,685 variant-affected genes were protein-encoding, of which 22,592 were flagged as non-synonymous mutations (Table S13).

### 3.9. Phylogenomic Analysis of the Origins of the MHR

**Figure 3:**
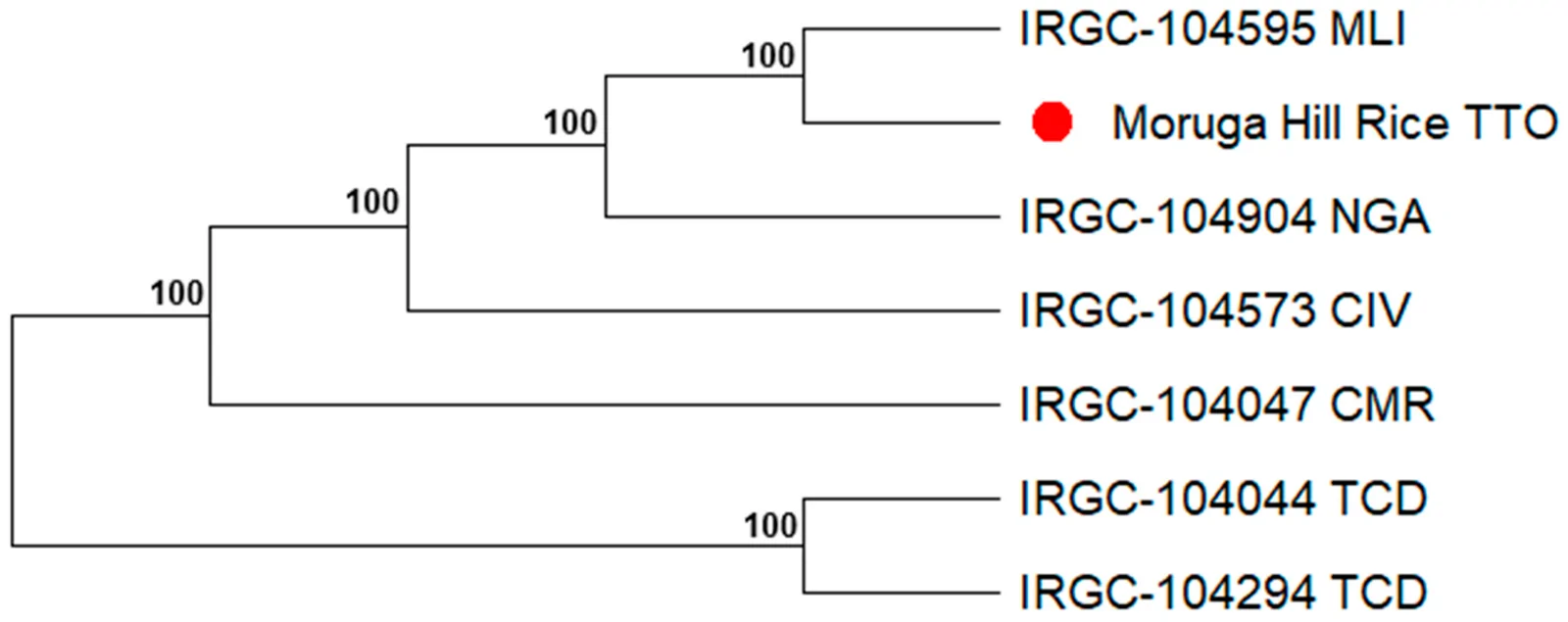
Phylogenomic subtrees estimating the closest origins of the MHR. Maximum-likelihood (ML) phylogenomic subtree (consensus) highlighting the Moruga Hill Rice TTO (red circle) clustering among five (5) African landraces. Bootstrap iterations = 1000. NGA—Nigeria; CMR—Cameroon; TCD—Chad; CIV—Cote d’ivoire; MLI—Mali; TTO—Trinidad and Tobago).

**Figure 4:**
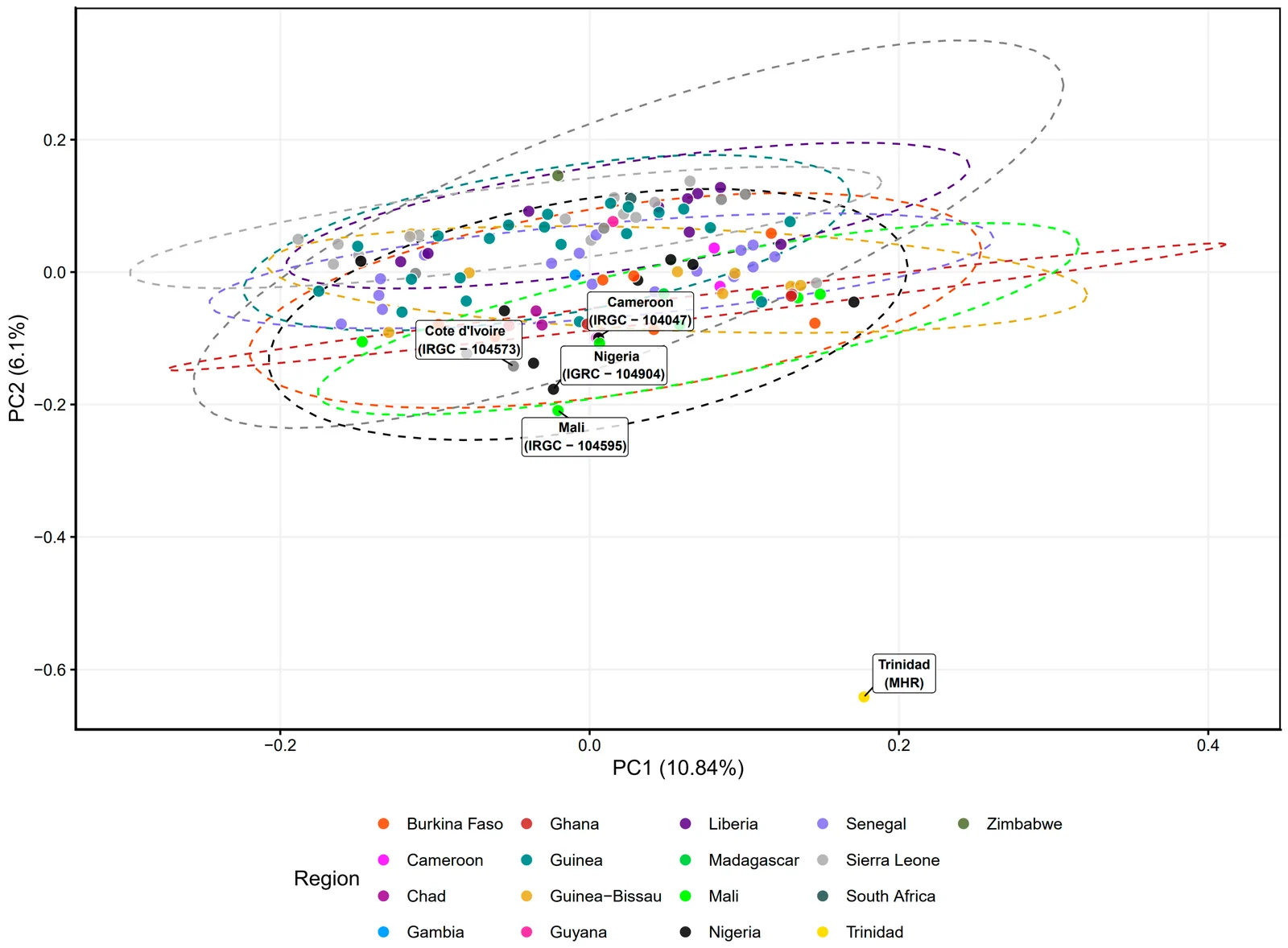
Comparison of the MHR, Trinidad sequence to 112 landraces through a principal component analysis (PCA) using the first two principal components. PCA shows close grouping of MHR to the IRGC−104595 (Mali) and IRGC−104904 (Nigeria) landraces.

**Figure 5:**
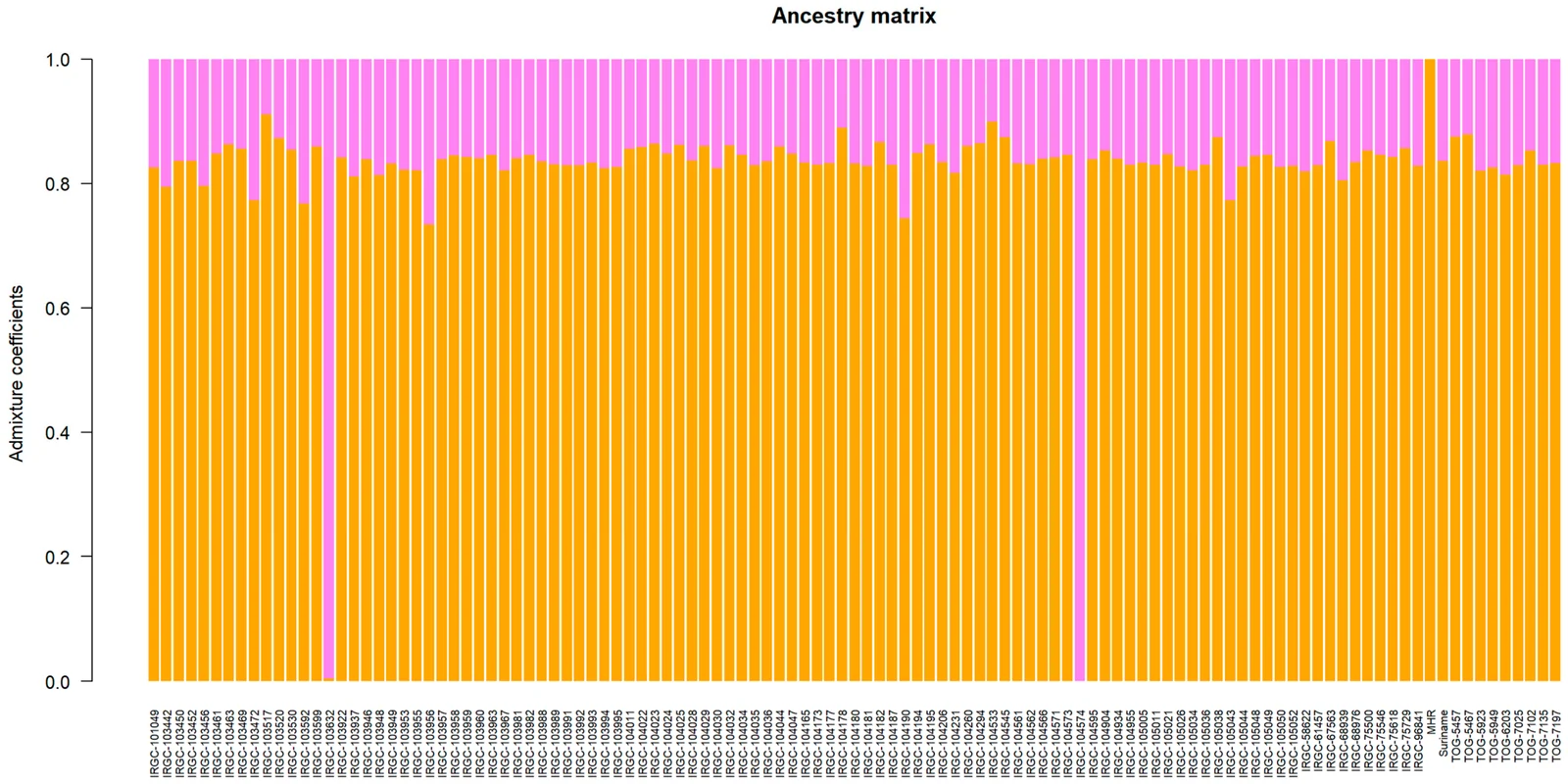
Population genetic structure of Moruga Hill Rice (MHR) compared to 112 African rice accessions. Ancestry coefficient matrix inferred using *sNMF* from genome-wide SNP data at *K* = 2. MHR exhibits high genetic homogeneity and clustered predominantly with one major ancestry component, indicating high genetic similarity and primary ancestry with West African *Oryza glaberrima* accessions.

After filtering, 62,646 high-quality SNP markers across 113 accessions were retained for phylogenomic analysis (Figure 3 and Figure S10). The maximum likelihood tree revealed MHR to be most related to the IRGC−104595 Malian accession. The PCA also placed the Trinidad MHR as most closely associated with IRGC−104595 (Mali) and IRGC−104904 (Nigeria) accessions; however, as a distinct distant cluster (Figure 4). Admixture coefficient estimation (*K* = 2) using *sNMF* also portrayed MHR as having high homogeneity (Figure 5). Nucleotide diversity for MHR (π = 0.29) was found to be significantly lower than all other landraces (π ≥ 0.40) (Table S14). Due to the presence of only one representative sample per country for MHR and select accessions, Nei’s heterozygosity could not be calculated.

## 4. Discussion

This study presents the first whole genome draft assembly of Moruga Hill Rice using a hybrid sequencing strategy that combines long-read contiguity with high-accuracy short reads. Reference-based scaffolding implemented in the MHR assembly can reduce the overall sensitivity to detect true structural variants (SVs), particularly large insertions, inversions, translocations or novel sequences not represented in the reference genome. As such, the scaffolded assembly should be interpreted as a reference-anchored representation rather than a fully de novo reconstruction of the genome structure. Downstream analyses were interpreted with this constraint in mind, and SNP-based analyses are less affected by this bias compared to SV discovery. Future de novo telomere-to-telomere assemblies incorporating ultra-long reads and Hi-C data for chromosome-scale scaffolding could improve detection of large structural variants by spanning repetitive regions, resolving complex genomic rearrangements, and capturing potential sequences absent from the current reference-guided assembly [69]. These approaches would enhance assembly continuity and provide a more complete representation of the MHR genome architecture, including chromosome organization and structural variation.

ONT long read sequencing of the MHR genome achieved genome coverage and N50 lengths comparable to assemblies of *O. sativa* indica [70]. BUSCO analysis indicated high completeness, though slightly lower than the reference genome. The MHR genome assembly (~372.9 Mb) was approximately 18% larger than the *O. glaberrima* OglaRS2 reference assembly (316 Mb). MHR contained a higher gene count than OglaRS2 (34,185 genes) but was more comparable to resequenced cultivars CG14, TOG5681, and G22 (49,662–51,262 genes) despite having a 20–25% larger genome [71]. The MHR assembly repeat content (50.34%) was much greater than the original CG14 genome assembly [72] but was found to be comparable to other *O. glaberrima* genomes [62,73]. Overall, this suggests that the increased genome size may reflect some structural variation or repetitive sequence expansion rather than major increases in coding gene content. Additionally, a larger genome size and gene content, as seen in the MHR, are often associated with gene duplication events, including tandem and segmental duplications that contribute additional gene copies and larger genomic regions [74,75,76]. However, overpredicted gene counts may also arise from pseudogenes or fragmented annotations, rather than representing entirely functional genes [77] due to the lack of MHR-specific transcriptome evidence.

The observed variation in chromosome sizes was consistent with the expected range for rice genomes [78]. The larger chromosome sizes observed in MHR relative to the reference are likely attributed to increased repeat content, additional assembled sequence, and accession-specific genomic differences [79,80]. Although chromosome 12 contained the highest gap proportion, it was not uniquely repeat-rich, suggesting that gap accumulation was influenced not only by overall TE abundance, but also by the presence of complex repeat structures, such as long TE arrays and highly similar repetitive sequences. These unresolved regions may introduce localized biases in chromosome-level analyses, including gene counts and gene density estimates, which should therefore be interpreted with consideration of assembly completeness.

The MHR’s high proportion of homozygous variants found (94.94%) was likely associated with the predominantly self-pollinating nature of *O. glaberrima* and its historical demographic background [81]. This pattern was unlikely to be solely caused by the propagation protocol, as multiple plants were maintained and seeds were bulk harvested to minimize genetic drift. The observed homozygosity may reflect reduced genetic diversity associated with historical founder effects following the introduction of MHR into Trinidad [82]. However, this hypothesis requires validation using population-level genomic analyses.

Interestingly, the MHR genome exhibited a relatively high SNP density (6.42 SNPs/kb) compared with previously reported *O. glaberrima* genomes (2.3–2.4 SNPs/kb) [71] but was comparable to values reported for some *O. sativa* genomes (~7.0 SNPs/kb) [83]. This elevated SNP density indicates a greater number of detected polymorphic sites within the MHR genome relative to available reference genomes. However, as this analysis was based on a single MHR accession, it should not be interpreted as a direct estimate of population-level nucleotide diversity. While local environmental pressures may have contributed to the retention of certain alleles, evidence for adaptive selection would require additional analyses, such as population differentiation or selection scans [84,85], which are beyond the scope of this study. Although technical factors, including sequencing depth, reference genome choice, and variant calling approaches, can influence SNP estimates, the use of the widely adopted *GATK* variant calling pipeline and stringent filtering criteria supports the reliability of the identified variants. This diversity observed in MHR could potentially harbour novel alleles associated with agronomic traits of interest. Comparative genomic and functional validation studies are thus required to substantiate these prospective applications.

The reference-based SNP calling approach, which relies on alignment of MHR reads to the OglaRS2 assembly, may bias estimates of genomic diversity by limiting variant discovery to regions present and reliably aligned in the reference assembly, thereby potentially excluding variation within divergent or reference-absent regions. Considering this limitation, the phylogenomic analysis placed MHR with strong support among West African landraces, showing closest affinity to accessions from Nigeria, Mali, Côte d’Ivoire, Ghana, and Cameroon. Although most closely related to the Malian landrace IRGC−104595, MHR remains genetically distinct. The low nucleotide diversity observed in MHR (π = 0.29), together with its high genetic homogeneity in *sNMF* analysis, suggests reduced genetic variation within the sequenced accession and may suggest a possible founder effect [82,86] associated with its introduction and subsequent cultivation in Trinidad. Confirmation of this hypothesis will require population-level sampling of multiple MHR accessions and comparative analyses of genetic diversity. Nevertheless, the strong genetic affinity between MHR and West African landraces, particularly the Malian IRGC104595 landrace, is consistent with historical records indicating that MHR was introduced to Trinidad by Merikins originating from regions including the Slave Coast region [6,86,87,88,89].

The current genome annotation was generated using an evidence-guided ab initio approach implemented in *Augustus*, incorporating extrinsic evidence from *O. glaberrima* cDNA and protein sequences, providing a first-pass gene set in the absence of MHR-specific RNA-seq data. Additionally, phenotypic evidence for biotic and abiotic stress tolerance has yet to be generated for the MHR. As a result, the overall confidence in this predicted gene set was constrained, particularly for genes putatively implicated in biotic and abiotic stress tolerance or disease resistance, where functional assignment remains provisional in the absence of transcriptional support. Some predicted loci may therefore represent pseudogenes or artifacts of the prediction process rather than truly expressed genes. However, the MHR genome assembly can be further improved by incorporating transcriptome-based evidence from multiple tissues and developmental stages to refine gene model accuracy through improved annotation of exon–intron boundaries, alternative splice isoforms, and tissue-specific gene expression. Transcriptome analyses of drought, salinity, and pathogen-stressed MHR tissues (leaves, roots, and panicles) will further improve genome annotation, validate candidate stress-related genes, and reveal tissue-specific and stress-responsive regulatory networks underlying adaptation and disease resistance.

Some key ontologies known to be associated with key agronomic genes were also represented among the variant-affected protein-coding genes. Chen et al. (2024) reported several differentially expressed genes (DEGs) annotated with the “defense response to bacterium” ontology when analyzing the transcriptomes of *Xoo-infected* rice lines [90]. Similarly, several variant-affected genes in MHR were associated with disease resistance-related ontologies that may have potential roles in defense responses; however, further investigations are required to determine their association and roles in disease resistance. Ontologies of variant-affected genes in the MHR associated with abiotic stress tolerance have all been reported in previous drought and salt stress studies performed on rice, all indicating DEGs possessing such ontologies [91,92,93]. These initial findings are consistent with previous studies reporting that the *O. glaberrima* genome contains numerous genes associated with abiotic stress tolerance [94,95]. While MHR transcriptome analysis has not been performed, these findings suggest that such variants may potentially affect pathogen recognition, immune signalling, and defense response pathways, and may represent putative loci related to disease resistance. Likewise, variants affecting genes associated with abiotic stress responses may affect tolerance levels to such conditions.

The seven most abundant TF families identified in MHR have been widely associated with stress tolerance and disease resistance in rice [96,97,98]. Preliminary evidence revealed multiple predicted TFs with homology to known regulators of abiotic stress responses, defense signalling, and pathogen resistance. As such, their potential regulatory roles in previously characterized genes need to be further studied.

Candidate blast and blight disease resistance genes in MHR were identified based on NLR orthology and NBARC domain phylogeny. Phylogenetic and RBH analyses provided complementary evidence for identifying candidate resistance gene relationships. Ten MHR NLR proteins showed phylogenetic affinity to previously characterized blast resistance proteins, including Pi-ta, Pi36, Pia, Pid3, Piz(t), and Pi37. Chr11_g1288 and Chr6_g2174 showed strong phylogenetic support and high sequence similarity to Pia and Pid3, respectively, supporting their classification as putative candidate proteins. Chr8_g570 clustered within the Pi-ta/Pi36 lineage and showed high similarity to Pi36, while additional RBH analyses identified MHR orthologs to previously characterized blast and bacterial blight resistance proteins, including Pi, Pik, Pi21, Xa13, Xa23, and Xa25 families. However, as most characterized resistance genes used for comparison originate from *O. sativa*, NLR orthology in these cases does not necessarily indicate conserved resistance function in *O. glaberrima*, and by extension MHR. Differences in their allelic variation, genomic context, and pathogen recognition specificity among NLRs can ultimately influence resistance phenotypes and effector recognition outcomes [99,100]. Discrepancies reported between RBH and phylogenetic placement likely reflect differences in the sequence regions analyzed. Phylogeny was based on conserved NB-ARC domains, which are useful for assessing evolutionary relationships among NLRs but may not fully capture divergence across other functional regions of the protein [101]. In contrast, RBH analysis incorporated full-length protein sequences, where conserved and divergent regions collectively influence similarity estimates. Therefore, discordance between these approaches may reflect domain-specific conservation, structural diversification, or lineage-specific evolution among NLRs. Consequently, these should be considered as putative resistance loci rather than confirmed functional resistance genes. As such, functional validation through pathogen challenge assays, expression profiling, transient assays, and targeted gene knockouts will be required to confirm their roles in disease resistance in the MHR.

An extensive number of pathogenesis-related (PR) proteins were reported as PR-1, PR-3 (chitinases and precursors of chitinases), and PR-5 (thaumatin-like proteins (TLPs)). Acidic endochitinases and xylanase inhibitors were also noted across the genome, some of which were previously characterized as having chitinase activity. These PR-proteins and chitinases have been widely associated with antifungal defense mechanisms, including chitin degradation, and may represent putative resistance genes against blast and RSB diseases [102,103].

Numerous TLPs with in vitro antifungal activity and roles in plant defense reported in the literature were also annotated in the MHR genome [104,105]. Given that rice TLPs are strongly induced by pathogens like *Xoo*, and overexpression of a TLP *PR-5* gene enhances tolerance to *R. solani* [104], the high number of these predicted genes in MHR suggests the presence of loci potentially associated with similar plant defense responses.

Screening the MHR annotated genes revealed several homologs to disease resistance genes, such as *Pikm* and *Pib/Pi-ta* classes [106]. *Pikm*-specific resistance typically requires both *Pikm1* and *Pikm2* genes to be located in an adjacent manner within the *Pik-m* locus [107]. Notably, MHR *Pik6-NP* and *Pik-2*-like genes (both *Pikm* alleles) were placed on separate chromosomes rather than within a locus, thus suggesting potential inaccuracies in gene placement or DNA transposons facilitating gene shuffling [108]. The MHR would benefit from telomere-to-telomere de novo assembly to resolve structural variants such as these. Further conformational analyses are also needed to determine the functional roles of MHR *Pikm* alleles. MHR gene homologs belonging to the *Pib* and *Pi-ta* families have been extensively characterized in previous studies for broad-spectrum disease resistance against *M. oryzae* [109]. *Pi-ta* encodes an NBS-LRR protein that specifically recognizes the *M. grisea* avirulence effector Avr-Pita, triggering downstream defense signalling cascades [110]. Genomic analysis revealed a *Pib* locus on MHR chromosome 2 containing four annotated gene sequences: a *Pib* variant, *Pib*-partial, *Pib-CO*, and *Pib-GLA*. Functional annotation analysis indicated that *Pib-CO* and *Pib-GLA* possess gene ontology terms associated with defense responses, suggesting potential roles in pathogen recognition or downstream signalling pathways. The identification of *Pib* and *Pi-ta* resistance gene family homologs in the MHR genome provides evidence of conserved resistance-associated loci that may contribute to ETI-related defense responses against *M. oryzae*. However, their contribution to blast resistance remains to be experimentally validated [111,112].

The MHR genome contained a rich repertoire of Resistance Gene Analogs *(RGAs*) among other defense-related genes, suggesting strong potential for resistance against various pathogens. MHR *RGA1* gene homolog encodes the Gα subunit of the heterotrimeric G protein critical for salt, cold, and drought stress tolerance in rice [113,114]. Homologs of MHR *RGA4* and *RGA5/RGA5*-like genes encoding NB-LRR proteins demonstrated dual recognition specificity and function as essential components in the detection of *M. oryzae* effectors AVR1-CO39 and AVR-Pia to activate ETI pathways [115]. Previous functional studies have established *RGA3* as a key determinant of blast disease resistance in rice cultivars [116]. *RGA3* genes were widely distributed throughout the MHR genome. However, transcriptomic analysis is required to accurately identify and characterize these candidate genes and their pathogen resistance capacity. A *Pi63*-like gene on chromosome 4 was placed within a cluster structurally resembling the Kahei cultivar *Pikahei1(t)* QTL (chromosome 4) [116]. Several *RGAs* were predicted within a ~452 kbp cluster upstream and downstream of *Pi63* from chromosomal position 29,800,000 to 30,251,691. When accounting for interspaced transposable elements, this MHR gene cluster shares some structural resemblance to that of the ~300 kb *Pikahei1(t)* QTL. These findings prompt further investigation into this MHR gene cluster to determine its similarity to the *Pikahei1(t)* QTL and its involvement in response to rice blast fungal infection.

Genomic analysis revealed the presence of a gene sequence exhibiting high homology to *Pi54*, a well-characterized broad-spectrum blast resistance gene. MHR defense-related *RPM1* and their homologous sequences, which have been previously associated with resistance mechanisms against *M. oryzae*, *Xoo*, and sheath blight pathogens [117]. The MHR also contained genes encoding *RPP13*-like and *RPP8*-like proteins, both of which have been functionally linked to *Xoo* resistance responses [118]. Mildew resistance Locus O (MLO)-like proteins are involved in various stress responses, and genes from the *Pyricularia oryzae* Resistance 21 (*Pi21*) and *Xa* families were also predicted. Specifically, *Pi21* is known for partial durable blast resistance in its recessive form [116,119,120]. *SWEET11/Xa13* confers bacterial blight resistance as a recessive *xa13* [121,122], while the receptor kinase *Xa21* confers broad-spectrum *Xoo* resistance [123], both of which were predicted in the MHR genome. The presence and distribution of these diverse defense-related genes highlight the MHR genome as a valuable resource for studying and potentially enhancing disease and stress resistance in rice.

Numerous candidate genes with potential roles in abiotic stress tolerance were identified based on sequence homology and GO annotation, including candidates associated with salt stress and drought/water deprivation responses. These included small heat shock proteins (sHSPs), ABA receptors (PYLs), DREB transcription factors, aquaporins (PIP1-1, PIP1-2, PIP1-3), dehydrins (Rab proteins), CPKs, MAPKs, and stress-related enzymes such as SOD and 6PGDH. Many of these genes have documented roles in tolerance to drought, salinity, cold, and oxidative stress [124,125]. Although expression data are lacking, their presence suggests potential genetic capacity for responses to abiotic stress.

A large number of HSPs and heat shock transcription factors were identified, including members of groups A and C that are constitutively expressed during heat stress in *O. sativa* [126]. Multiple HSP classes function as molecular chaperones under abiotic stress conditions [126,127,128]. Numerous small HSPs (classes I, II, III, and VI) and mitochondrial HSPs were also predicted in MHR, many of which have been previously associated with heat and drought responses in other plant systems. Their presence suggests potential involvement in abiotic stress response pathways requiring functional validation to determine their specific roles in MHR [129,130,131,132].

In contrast, fewer cold-related genes were identified. Among these, *OsTRXH1* is induced by cold, ABA, and salt stress [133], while *OsWRKY45* alleles regulate drought and cold responses in rice [134,135]. Sixteen peroxidase genes associated with oxidative stress responses were also detected, several showing duplication and homology to genes involved in salt and drought tolerance. These findings support potential tolerance to oxidative damage under multiple stress conditions [136].

MHR represents a unique and historically important African rice germplasm preserved in the Caribbean for over two centuries. The genomic insights generated in this study provide an important foundation for understanding its evolutionary history, genetic distinctiveness, and potential agronomic value. These findings also expand the limited genomic resources currently available for African rice landraces and support their future conservation and utilization in breeding programs. This current study presents the first draft genome assembly of the *O. glaberrima* Moruga Hill Rice grown in Moruga, Trinidad. While we only report on the potential of genes associated with disease and abiotic stress responses based on homology and ontologies, it warrants further analyses, such as transcriptome studies to demonstrate their expression in response to various biotic and abiotic stresses, and the extent to which these genes contribute to resistance or stress tolerance in MHR.

## Figures and Tables

**Table 1:** Summary of Illumina and ONT read sequences used for whole genome assembly.

Sequencing Platform	Flowcell ID	No. of Reads	N50 Read Length (kbp)	No. of Bases (Gbp)	Estimated Genome Coverage (×)
Illumina Novaseq X Plus (PE150bp)	22GF7HLT3	317,401,980	0.15	47.60	137.06
Oxford Nanopore Technologies (ONT) GridION X5	FAX82026	981,340	15.55	8.69	25.02
	FAW80308	385,360	13.02	2.80	8.06

**Table 2:** Comparison of Illumina read mapping statistics, sequencing depth, and alignment error profiles before and after genome polishing.

	Before Genome Assembly Polishing	After Genome Assembly Polishing
Mapping rate (%)	99.43	99.20
Properly paired rate (%)	97.90	98.02
General error rate (%)	0.87	0.16
Insertion rate (%)	0.185	0.003
Deletion rate (%)	0.039	0.003
Mapped reads with at least one insertion	21.19	0.47
Mapped reads with at least one deletion	5.11	0.46

**Table 3:** Summary of the MHR genome assembly statistics.

Category	Metric	Value
Genome assembly (contigs)	Assembled genome size (bp)	372,919,726
	No. of contigs	1333
	Contig N50 (kbp)	709.263
	Contig L50	153
	Contig N90 (kbp)	147.116
	Contig L90	576
	Maximum Contig Length (kbp)	3209
	BUSCO score (Embryophyta; 100% = 1614)	99.0%
	BUSCO score (Liliopsida; 100% = 3236)	97.0%
	BUSCO score (Poales; 100% = 4896)	98.0%
Genome scaffolds	Scaffolded genome size (bp)	379,731,140
	Placed contigs	1203
	Unplaced contigs	130
	Scaffold N50 (kbp)	30,618
	Scaffold L50	6
	Scaffold N90 (kbp)	25,052
	Scaffold L90	11
	Average Scaffold Length (bp)	2,655,463
	Maximum Scaffold Length (kbp)	43,640
	Total length of placed contigs (N’s inclusive; bp)	377,490,348
	Total length of unplaced contigs (bp)	2,240,792
	Genome gaps (bp)	6,811,414
	Genome gap percentage	1.794%
	BUSCO score (Embryophyta; 100% = 1614)	98.9%
	BUSCO score (Liliopsida; 100% = 3236)	96.7%
	BUSCO score (Poales; 100% = 4896)	97.4%
	GC content (%)	43.58

**Table 4:** Summary of the MHR chromosomal sizes compared to the *O. glaberrima* GCF_000147395.1 reference assembly.

Chromosome	Size (Contigs) (bp)	Scaffolded Size (bp)	Size of Gaps (bp)	*O. glaberrima* Reference (bp)
Chr1	43,199,523	43,639,535	440,012	39,656,875
Chr2	37,007,463	37,454,331	446,868	34,451,706
Chr3	37,293,192	37,592,902	299,710	35,526,804
Chr4	32,954,945	33,735,904	780,959	31,572,834
Chr5	28,733,432	29,329,287	595,855	26,274,045
Chr6	31,109,974	31,670,030	560,056	29,275,208
Chr7	30,048,122	30,618,242	570,120	26,599,614
Chr8	27,830,779	28,263,788	433,009	25,472,747
Chr9	24,684,205	25,052,276	368,071	21,796,211
Chr10	23,709,088	24,397,572	688,484	22,418,184
Chr11	29,566,986	30,094,219	527,233	26,393,634
Chr12	24,483,487	25,562,768	1,079,281	25,020,143
Plastid	58,038	79,494	21,456	132,629
Unplaced	2,240,492	2,240,792	300	2,730,412
Total	372,919,726	379,731,140	6,811,414	347,321,046

**Table 5:** Classification of transposable elements identified in the MHR genome draft assembly.

Class		bpMasked	%Masked
LINE			
LINE-L1		176,300	0.05%
LINE-unknown		5,075,394	1.36%
LTR			
LTR-Copia		13,498,242	3.62%
LTR-Gypsy		72,186,824	19.36%
LTR-TRIM		586,608	0.16%
LTR-unknown		1,818,697	0.49%
SINE			
SINE-unknown		1,114,584	0.30%
TIR			
MITEs	TIR-CACTA	14,411,999	3.86%
	TIR-Mutator	20,305,032	5.44%
	TIR-PIF_Harbinger	10,591,854	2.84%
	TIR-Tc1_Mariner	15,996,877	4.29%
	TIR-hAT	5,760,764	1.54%
TIR-PILE		984,494	0.26%
TIR-POLE		476,186	0.13%
TIR-unknown		676,882	0.18%
Centromeric_repeat		1,684,353	0.45%
nonLTR			
nonLTR-ERTBV		327,158	0.09%
nonTIR			
nonTIR-helitron		21,326,980	5.72%
rDNA			
rDNA-45S		108,079	0.03%
repeat_fragment		408,349	0.11%
satellite_DNA		225,787	0.06%
Total interspersed		187,741,443	50.34%
snRNA		8127	0.0%
Total		187,749,570	50.35%

**Table 6:** Reciprocal best BLAST hits (RBH) results on MHR protein sequences (E-value cutoff = 1.0 × 10^−5^).

NLR Proteins	NLR Class	MHR Protein Length (aa)	RBH Description	Similarity (%)	Alignment Coverage (%)	E-Value
Chr8_g570	CNL	1005	Pi36	85.37	99	1.0 × 10^−197^
Chr11_g1288	CNL	969	Pia-C_2	92.02	99	0
Chr6_g2174	CNL	899	Pid3	96.54	100	0
Chr1_g5335	CNL	1174	Pi37	84.44	74	1.0 × 10^−7^
Chr4_g2509	-	247	Pi21	98.38	100	0
Chr5_g1843	-	524	Pik-2	80.34	50	1.0 × 10^−5^
Chr6_g2238	-	845	Pik-2	99.76	100	0
Chr11_g4069	-	269	Pi54	73.41	74	1.0 × 10^−5^
Chr5_g19	-	629	Xa21	70.40	52	1.0 × 10^−5^
Chr5_g126	-	106	xa5	96.22	100	0
Chr6_g3734	-	119	Xa27	88.89	85	1.0 × 10^−24^
Chr8_g4137	-	307	xa13	100	100	0
Chr10_g1245	-	1094	Xa3	70.33	99	1.0 × 10^−154^
Chr11_g3558	-	98	Xa23	100	87	0
Chr11_g4478	-	651	Xa4	84.95	100	1.0 × 10^−200^
Chr12_g2279	-	293	Xa25	97.30	100	0

## Data Availability

The data presented in this study are openly available in Zenodo (https://doi.org/10.5281/zenodo.20140274). All ONT long-read and Illumina short-read sequences associated with the reported MHR genome assembly have been deposited at the Sequence Read Archive (SRA) in NCBI under BioProject PRJNA1224456. The MHR genome assembly FASTA file has been submitted to NCBI.

## Supplementary Materials

The supporting information can be downloaded from Zenodo at https://zenodo.org/records/21732528, accessed date 22 July 2026. Supplementary File S1 contains Figures S1–S11 and Tables S1, S2 and S5. Figure S1: BUSCO analysis on the MHR assembly on Embryophyta, Liliopsidia and Poales databases; Figure S2: GenomeScope k-mer profile of the Moruga Hill Rice (MHR) genome. K-mer frequency distribution (k = 19). The estimated haploid genome length is ~357 Mb, with ~0.0915% heterozygosity, ~0.071% read-error rate, and ~66.3% unique sequence content; Figure S3: Transcription factor (TF) counts across 56 TF families of *O. glaberrima* in the MHR; Figure S4: Transcription factor (TF) counts distribution across the MHR chromosomes and pseudomolecules; Figure S5: Illumina read-depth distribution and cumulative coverage before genome polishing and gap-filling; Figure S6: Illumina read-depth distribution and cumulative coverage after genome polishing and gap-filling; Figure S7: Canonical NLR gene classifications and counts found using the Resistify pipeline for each chromosome of the MHR genomes; Figure S8: Phylogenetic analysis of the 474 canonical NB-ARC domains in MHR against cloned blast resistance genes; Figure S9: Phylogenetic analysis of the 474 canonical NB-ARC domains in MHR against cloned blight resistance genes; Figure S10: Maximum likelihood (ML) phylogenomic tree highlighting the closest ancestral landrace of the MHR. Bootstrap iterations = 1000; Figure S11: Bioinformatics workflow for the de novo genome assembly, annotation, and comparative analysis of Moruga Hill Rice (*Oryza glaberrima*); Table S1: List of cloned blast disease resistance genes used to determine the phylogenetic relation of MHR NLR genes; Table S2: List of cloned blight resistance genes used to determine the phylogenetic relation of MHR NLR genes; Table S3: Gene Ontology counts for mining potential genes associated with disease resistance and stress tolerance across the MHR genome; Table S4: Rice accessions used for phylogenomic analysis in this study; Table S5: Summary of annotated gene counts across chromosomes and pseudomolecules of the MHR genome; Table S6: Transcription factor (TF) counts across 56 TF families of *O. glaberrima* in the MHR; Table S7: Transcription factor (TF) counts distribution across the MHR chromosomes and pseudomolecules; Table S8: Transposable element counts and proportions across the MHR genome; Table S9: MHR NLR candidates identified by Resistify, including motif structure, domain classification, and NBARC motif content; Table S10: Distribution of NLR gene classes identified by Resistify across chromosomes in the MHR genome; Table S11: Summary of the MHR genomic variant statistics; Table S12: SNPeff genomic variant effects results; Table S13: Variant affected protein-coding genes in the MHR genome; Table S14: Nucleotide diversity (π) estimates for *O. glaberrima* landraces used in the population comparison.

## Abbreviations

The following abbreviations are used in this manuscript:

ABA	Abscisic acid
AVR	Avirulence
BUSCO	Benchmarking Universal Single-Copy Orthologs
CDPK	Calcium-dependent protein kinase
CDS	Coding sequence
CN	Coiled-coil nucleotide-binding
CNL	Coiled-coil nucleotide-binding leucine-rich repeat
CTAB	Cetyltrimethylammonium bromide
DP	Depth of coverage
DREB	Dehydration-responsive element-binding
ETI	Effector-Triggered Immunity
GO	Gene ontology
HRM	His Royal Majesty
HSP	Heat shock protein
InDel	Insertion & Deletion
LAI	LTR Assembly Index
LD	Linkage disequilibrium
LINE	Long interspersed nuclear element
LTR	Long Terminal Repeats
MHR	Moruga Hill Rice
MITE	Miniature inverted-repeat TEs
MQ	Mapping quality
NB-ARC	Nucleotide-binding adaptor shared by APAF-1, R proteins and CED-4
NLR	Nucleotide-binding leucine-rich repeat
ONT	Oxford Nanopore Technologies
PCA	Principal component analysis
PIP	Plasma membrane intrinsic protein
PYL	Pyrabactin Resistance-like
QD	Quality by depth
QTL	Quantitative trait locus
QV	Quality value
*R*	Resistance gene
RBH	Reciprocal best BLAST hit
RLK	Receptor-like kinase
RLP	Receptor-like protein
sHSP	Small heat shock protein
SINE	Short interspersed nuclear element
SNP	Single Nucleotide Polymorphism
*T*	Tolerance gene
TE	Transposable element
TIR	Terminal inverted repeat
TNL	Toll/interleukin-1 receptor nucleotide-binding leucine-rich repeat
Ts/Tv	Transition/Transversion
